# Flexible Inkjet-Printed pH Sensors for Application in Organ-on-a-Chip Biomedical Testing

**DOI:** 10.3390/bios16010038

**Published:** 2026-01-03

**Authors:** Željka Boček, Donna Danijela Dragun, Laeticia Offner, Sara Krivačić, Ernest Meštrović, Petar Kassal

**Affiliations:** 1 Faculty of Chemical Engineering & Technology, University of Zagreb, Trg Marka Marulića 19, 10000 Zagreb, Croatia; zbocek@fkit.unizg.hr (Ž.B.); ddragun@fkit.unizg.hr (D.D.D.); laeticia.offner@bordeaux-inp.fr (L.O.); skrivacic@fkit.unizg.hr (S.K.); 2 École Nationale Supérieure de Matériaux, d’Agroalimentaire et de Chimie (ENSMAC), Bordeaux INP, 16 Avenue Pey-Berland, 33607 Pessac, France

**Keywords:** inkjet printing, pH sensor, electrochemical sensor, printed sensor, potentiometry, polyaniline, flexible sensor, lung-on-a-chip, biomedical testing

## Abstract

Reliable models of the lung environment are important for research on inhalation products, drug delivery, and how aerosols interact with tissue. pH fluctuations frequently accompany real physiological processes in pulmonary environments, so monitoring pH changes in lung-on-a-chip devices is of considerable relevance. Presented here are flexible, miniaturized, inkjet-printed pH sensors that have been developed with the aim of integration into lung-on-a-chip systems. Different types of functional pH-sensitive materials were tested: hydrogen-selective plasticized PVC membranes and polyaniline (both electrodeposited and dropcast). Their deposition and performance were evaluated on different flexible conducting substrates, including screen-printed carbon electrodes (SPE) and inkjet-printed graphene electrodes (IJP-Gr). Finally, a biocompatible dropcast polyaniline-modified IJP was selected and paired with an inkjet-printed Ag/AgCl quasireference electrode. The printed potentiometric device showed Nernstian sensitivity (58.8 mV/pH) with good reproducibility, reversibility, and potential stability. The optimized system was integrated with a developed lung-on-a-chip model with an electrospun polycaprolactone membrane and alginate, simulating the alveolar barrier and the natural mucosal environment, respectively. The permeability of the system was studied by monitoring the pH changes upon the introduction of a 10 wt.% acetic acid aerosol. Overall, the presented approach shows that electrospun-hydrogel materials together with integrated microsensors can help create improved models for studying aerosol transport, diffusion, and chemically changing environments that are relevant for inhalation therapy and respiratory research. These results show that our system can combine mechanical behavior with chemical sensing in one platform, which may be useful for future development of lung-on-a-chip technologies.

## 1. Introduction

The pH value is a parameter of huge importance and is purposefully tracked in environmental monitoring, the food and beverage industry, and various biomedical applications [1,2,3]. While a classical glass pH electrode remains the gold standard for commercialized use, more and more applications call for miniaturization, affordability, and ease of use, the demand for which will inevitably have to be answered in the near future. Replacing the fragile glass body, filled with a pressure and leakage-sensitive electrolyte, with a planar, miniaturized, and easily fabricated all-solid-state potentiometric system is becoming a necessity [4].

pH sensor miniaturization could greatly benefit from manufacturing techniques that support inline fabrication, processing, and modification of the electrodes to produce final sensors with as few steps as possible, preferably with a single fabrication method. Aside from miniaturization, it is important that the manufactured pH sensors overcome obstacles related to flexibility, invasiveness, performance characteristics (stability, sensitivity, response time), and accuracy. Moreover, since these sensors tend to be single-use and disposable, especially in biomedical applications, their production must provide low-cost sensors [5]. A trend of lowering sensor production costs by reducing material usage, energy consumption, and overall fabrication time has opened the door for additive manufacturing techniques to enter the sensor field. Although screen-printed miniaturized solid-state sensors still dominate commercially, inkjet printing is rapidly gaining attention in the research community [6,7]. Inkjet printing is a contact-free deposition technique in which picoliter droplets are precisely ejected onto a substrate, forming continuous films or patterns as they dry. This approach enables higher printing resolution compared to screen-printing and allows for patterning on prefabricated or sensitive structures that would be incompatible with contact-based screen printing [8]. Inkjet printing was thus successfully used in the development of several solid-state potentiometric ion-selective electrodes, such as potassium-selective electrodes [9], ammonium-selective electrodes [10,11], sulfide-selective electrodes [12], nitrate-selective electrodes [13], and pH-sensitive electrodes [1,5].

Two-electrode electrochemical sensors, mostly represented by potentiometric sensors, are primarily simpler to fabricate compared to three-electrode electrochemical sensors. The exception of the counter electrode is justified by the working principle of the potentiometric method, which is based on measurements of the equilibrium phase boundary potential using a voltmeter with high input impedance [14,15]. As such, these measurements do not consume the sample, have extremely low energy, and minimal sample preparation requirements [16]. Along with electrodes, modern readout devices are being miniaturized, thus enabling true integration into various environments, including portable and wearable devices [17].

Polymeric ion-selective membranes (ISMs) containing an ionophore are well-described in the literature, and hydrogen-selective electrodes (H-ISEs) are no exception [2]. The membrane usually comprises PVC dissolved in an organic solvent with the addition of a plasticizer, an ion exchanger molecule that enables reversible ion partitioning between the organic (ISM) and aqueous (solution) phases, and the ionophore, the component that selectively and reversibly complexes the analyte ion [18,19]. However, sustainability and toxicity concerns, both over the use of organic solvents and due to the leaching of membrane components, hinder the application of H-ISEs for biomedical purposes [20].

In contrast, polyaniline (PANI) is widely regarded as a biocompatible [21], pH-sensitive electrode material [22,23]. It exists in three redox forms: fully reduced leucoemeraldine, partially oxidized emeraldine, and fully oxidized pernigraniline. The emeraldine form of PANI reversibly changes between two forms, depending on the pH–emeraldine base (PANI-EB) exists in alkaline environments, while emeraldine salt (PANI-ES) is a conductive form more stable in acidic environments [23,24,25]. This reversible pH-dependent change, when paired with a reference electrode, can be used for potentiometric pH monitoring, as this transition results in a potential change exhibiting Nernstian sensitivity of about 60 mV/pH [22], while higher sensitivity can be achieved using specific dopants or improving the delocalization of π-electrons [5,22]. PANI is most commonly deposited using electrodeposition methods, as they result in homogeneous, pure, and mechanically stable layers [1]. However, this approach is not easy to scale up and integrate with mass manufacturing methods [26,27]. Moreover, the doping anion used during electrodeposition also affects the pH sensing properties of PANI, with bulkier anions suppressing the pH sensitivity, while smaller anions give rise to a supernernstian response [23].

A potentiometric system is not complete without a reliable reference electrode. From a technical standpoint, fabricating a reference electrode via inkjet printing follows essentially the same workflow as ISE production. The process consists of printing silver conductive tracks and forming the AgCl layer, either by chemical or electrochemical oxidation [28,29], thus obtaining a quasi-reference electrode (qRE). The fabrication of such electrodes is quite fast and simple, and they provide a stable potential in a constant chloride environment.

Reliable models of the lung environment are important for research into inhalation products, drug delivery, and how aerosols interact with tissue. pH fluctuations frequently accompany real physiological processes in pulmonary environments, so monitoring pH in such systems is of great importance. Local pH variations play a critical role in pulmonary physiology and pathophysiology [30] and are increasingly recognized as an important parameter in advanced in vitro lung models [31,32,33]. In the context of drug delivery through inhalation, the pH of the airway surface liquid directly influences drug dissolution, chemical stability, ionization state, and consequently, the local bioavailability of inhaled therapeutics [34]. This is particularly relevant for weakly acidic or basic drug molecules, where small pH changes may significantly alter their pharmacokinetic and pharmacodynamic behavior at the site of action. In addition to drug-related aspects, pulmonary infections and inflammatory conditions are frequently associated with local acidification of the lung microenvironment. Infection-driven metabolic activity, immune cell activation, and altered epithelial transport processes can lead to measurable pH shifts, which serve as indicators of disease progression and tissue response [35]. Continuous monitoring of such changes is therefore of high clinical relevance, especially for studying infection-related acidosis and inflammatory lung diseases. Integrating pH sensors into lung-on-a-chip platforms enables real-time and spatially resolved monitoring of microenvironmental changes under dynamic and physiologically relevant conditions. Unlike conventional static cell culture systems, lung-on-a-chip models combined with embedded sensors allow the direct correlation of pH fluctuations with aerosol exposure, drug delivery events, and cellular responses [36]. This capability provides an important link between engineering design, pharmacological evaluation, and disease modeling, thereby supporting the development of more predictive in vitro tools for pulmonary applications [37].

In this work, we have studied several different types of printed polymeric potentiometric pH sensors, with the aim of their integration in an artificial lung model for biomedical testing; analytical performance, robustness, stability, and flexibility are key for this application. We have developed a hybrid lung-on-a-chip setup that combines an electrospun membrane that imitates the mechanical properties of the alveolar barrier with a hydrogel layer that behaves similarly to the natural mucosal environment. While many usual in vitro systems are static and cannot really mimic the dynamic mechanical and chemical conditions inside real alveoli [37,38], the electrospun membrane of our system provides porosity and flexibility, while the hydrogel provides a hydrated layer, imitating biological tissue. Lastly, flexible inkjet-printed PANI-based electrodes were integrated and used to monitor proton diffusion through the lung system in real time.

## 2. Materials and Methods

### 2.1. Chemicals and Materials

Hydrogen ionophore I, bis(2-ethylhexyl) sebacate (DOS), potassium tetrakis(4-chlorophenyl)borate (KTpClPB), polyaniline emeraldine base (PANI-EB, *M*_w_~20,000), aniline (99 wt.%), tetrahydrofuran (THF, ≥99.5 wt.%), cyclohexanone, and ethylene glycol (EG) were purchased from Sigma Aldrich, Burlington, MA, USA. Acetic acid (HAc, 99.5 wt.%) was purchased from T.T.T., Sveta Nedelja, Croatia. Polyvinyl chloride (PVC, high molecular weight) was obtained from Fluka, Buchs, Switzerland. Dimethylformamide (DMF), ammonium sulfate ((NH_4_)_2_SO_4_), and potassium dihydrogen phosphate (KH_2_PO_4_) were acquired from Gram-Mol, Zagreb, Croatia. Dichloromethane (DCM) was purchased from CARLO ERBA Reagents S.A.S., Val-de-Reuil, France. Sulfuric acid (H_2_SO_4_, 96%) and sodium nitrate (NaNO_3_) were purchased from Lach-Ner, Neratovice, Czech Republic. Sodium alginate was acquired from Thermo Fisher Scientific, Kandel, Germany. Iron(III) chloride hexahydrate (FeCl_3_ × 6 H_2_O), calcium chloride (anhydrous, CaCl_2_), sodium sulfate (Na_2_SO_4_), potassium chloride (KCl), and potassium hydrogen phosphate (K_2_HPO_4_) were purchased from Kemika, Zagreb, Croatia. Magnesium sulfate (99%, anhydrous, MgSO_4_) was obtained from Acros Organics BV, Geel, Belgium.

Graphene ink (900695) was purchased from Sigma Aldrich, Burlington, MA, USA. Nanosilver ink (DM-SIJ-3201) and a commercial diluent (DM-SIJ-3201-DT) were purchased from Dycotec Materials Ltd., Calne, UK. Polyimide sheets were obtained from DuPont, Wilmington, DE, USA. A commercial glassy carbon electrode was acquired from BASi, West Lafayette, IN, USA. Commercial screen-printed carbon electrodes (ED-S1PE-C10) were purchased from MicruX Technologies, Gijón, Spain. A double junction Ag/AgCl/3M KCl/3M KCl reference electrode was obtained from Metrohm, Herisau, Switzerland. Polycaprolactone (PCL, Capa™ 6800) was acquired from Perstorp, Malmö, Sweden. Clear Microfluidic Resin (Photopolymer Resin Ver. 7.0a) was purchased from CADworks3D, Concord, ON, Canada.

All chemicals were used as received without further purification. All solutions were prepared using double-distilled deionized water (MilliQ, Millipore, Burlington, MA, USA).

### 2.2. pH Sensor Fabrication and Characterization

#### 2.2.1. Inkjet Printing of Electrodes

The electrode drawings were created in CorelDRAW X8 Graphics Suite, Corel Inc., Austin, TX, USA, with a working electrode diameter of 3 mm. Graphene ink was used to print the inkjet-printed graphene electrodes (IJP-Gr) in 5 passes using a Dimatix DMP-2850 printer (Fujifilm, Tokyo, Japan), on a polyimide substrate. The printing process was preceded by dilution with cyclohexanone (20% *v*/*v*). The total volume of diluted ink in the cartridge was 1.5 mL. After printing, the electrodes underwent thermal (300 °C for 1 h) and photothermal treatment (IPL flashing with Xenon X1100 system, Xenon, Wilmington, MA, USA) to ensure the conductivity of the printed electrodes by removing stabilizers.

The Ag reference electrode was printed in a single pass using a nanosilver ink, previously diluted with a commercial diluent (40% *v*/*v*). The substrate was heated to 60 °C during printing. No additional post-printing sintering treatment was applied to the silver electrodes, but they were modified to ensure a stable reference potential. AgCl deposition on printed Ag substrate was conducted chemically (immersing into 0.1 M FeCl_3_ solution; AgIJP-CD) or electrochemically (applying a constant current of 2.5 mA in 3 M KCl; AgIJP-ED) by varying the duration of the experiment. A stable reference potential was provided by ensuring a constant chloride background in measuring solutions (*c*(KCl) = 0.2 M), which is the concentration of chloride in CaCl_2_, and this was used for alginate crosslinking.

#### 2.2.2. Hydrogen Selective Electrodes

Hydrogen selective electrodes (H-ISE) were prepared and characterized by modifying the electrodes with a plasticized PVC-based ion-selective membrane (H-ISM) containing Hydrogen ionophore I (1 wt.%), DOS (65.6 wt.%), PVC (32.8 wt.%), and KTpClB (0.6 wt.%). The membrane components were dissolved in a solvent mixture of THF and cyclohexanone (50:50 *v*/*v*). Deposition of the membrane cocktail was initially performed by dropcasting on different electrode substrates, including glassy carbon, screen-printed carbon, and inkjet-printed graphene, and then by automated jetting deposition (so-called spotting), using an automated dispensing system (Nordson E3 EFD, Westlake, OH, USA). The membrane was deposited by spotting 1–6 spots on SPEs, after which the electrodes underwent calibration in phosphate buffers of varying pH. Finally, IJP-Gr electrodes were modified with 4 spots of H-ISM and calibrated in the same way.

#### 2.2.3. Polyaniline Deposition Methods

Multiple deposition methods for modifying the electrode surface with polyaniline were tested, including electropolymerization (PANI-ED) from an aniline monomer solution (0.1 M aniline in 1 M H_2_SO_4_) and dropcasting of PANI-EB dispersion directly onto the electrode. Deposition methods were tested on different electrode substrates, including glassy carbon electrode (GCE), screen-printed carbon, and inkjet-printed graphene. PANI-ED electrodeposition was performed via cyclic voltammetry by scanning from −0.1 to +0.8 V at 10 mV/s, with a variable number of scans applied (10–16).

Electrodes were modified with PANI-EB by dispersing 1 mg/mL of PANI-EB in a mixture of THF:EG (90:10, *v*/*v*) and dropcasting it on the working electrode (SPE or IJP-Gr) in 1.5 µL aliquots while heating at 50 °C on a hotplate. Each layer was dried for at least 10 min until a final dropcast volume of 18 µL was deposited.

#### 2.2.4. Electrochemical Measurements

Analytical properties were evaluated through potentiometric measurements in phosphate buffers with pH values adjusted to the physiological range (pH 5.4–8). Potentiostatic measurements were conducted on Palmsens4, PalmsensBV, Netherlands. Potentiodynamic measurements were performed on Lawson EMF6.

Interference testing was performed according to an example in the literature, conducted on a similar system [4]. Potentiodynamic measurement of the open circuit potential of the PANI-EB pH-sensitive system was measured for 10 min in a buffer solution (pH = 6.4, 0.2 M KCl) without any interferents. Then, small volumes of a concentrated interferent solution (1 M of either NaNO_3_, Na_2_SO_4_, (NH_4_)_2_SO_4,_ or MgSO_4_ in a buffer solution pH = 6.4, 0.2 M KCl) were added to achieve a final interferent concentration of 1 mM, 5 mM, or 10 mM. For each addition, the open circuit potential was measured for 5 min.

### 2.3. Lung-on-a-Chip Model Fabrication

The lung-on-a-chip housing was modeled in Autodesk Fusion 360, incorporating a hexagonal cross-section, a curved, dome-like base to emulate alveolar walls, and lateral ports for controlled delivery or removal of gases or aerosols (Figure S1). The model was 3D printed using digital light processing (DLP) on a CADworks3D H50-405 printer (CADworks3D, Concord, ON, Canada) using Clear Microfluidic Resin.

To fabricate the membrane, the polymer solution was first prepared by dissolving 1 g of PCL in 10 mL of a solvent mixture composed of DMF and DCM in a 1:9 ratio. The solution was stirred at 350 rpm for 1 h at room temperature until a homogeneous solution suitable for electrospinning was obtained. The properties of the resulting fibers, including their diameter, porosity, and surface structure, depended on the solution’s characteristics, applied voltage, and flow rate [39]. This solution was then loaded into a syringe fitted with a 22G needle. Process parameters, optimized through prior experiments, were set to a voltage of 12 kV, a tip-to-collector distance of 12 cm, and a flow rate of 12 × 10^−3^ mL/min. Upon completion of electrospinning, the aluminum foil supporting the newly formed membrane was carefully removed from the collector and allowed to dry under ambient conditions for several hours.

To prepare the 2% sodium alginate solution, 2 g of alginate powder was introduced slowly in a thin stream directly into 100 mL of deionized water at room temperature under continuous stirring, to ensure full hydration of the polymer chains and prevent aggregation. The dispersion and hydration process continued for 60–90 min, until the mixture developed a viscous, pseudoplastic consistency. Although the solution was viscous, alginate requires ionic crosslinking, which was achieved by spraying 0.1 M CaCl_2_ to form a mechanically stable hydrogel.

The assembly of the lung-on-a-chip system began with securing the electrospun PCL membrane onto the 3D-printed housing, ensuring full mechanical contact between the membrane surface and the supporting frame. Once the membrane was fixed in position, 5 mL of the pre-prepared alginate solution was applied using a casting approach, allowing the hydrogel precursor to spread uniformly across the membrane surface. Crosslinking was then initiated by spraying 0.75 mL of a 0.1 M CaCl_2_ solution. After the initial hydrogel network was fully crosslinked, a PANI-modified pH sensor was carefully placed on top of the gel surface, and a second hydrogel layer (4 mL) was applied over it and subsequently crosslinked with 0.6 mL of 0.1 M CaCl_2_, creating a compact two-layer hydrogel architecture. The assembled system was then connected to the breathing source, which delivered a 10 wt.% acetic acid stream to test pH-dependent permeability. A schematic of the experimental setup is depicted in Figure S2 in the Supplementary Information.

## 3. Results and Discussion

### 3.1. Reference Electrode Optimization

Potentiometric measurements are usually conducted using a two-electrode system: the working electrode, whose potential changes with a change in the concentration of the analyte (in our case, a change in pH), and the reference electrode, the potential of which should remain stable and constant during the measurement.

To ensure a stable reference potential point in the final fully inkjet-printed system, silver inkjet-printed electrodes were modified chemically or electrochemically to deposit a layer of AgCl, forming a reversible Ag/AgCl redox pair. The stability of the reference point is crucial, as potential drift over time, induced by the potential dissolution of the thin AgCl layer, would introduce significant error into the measurement results.

**Figure 1:**
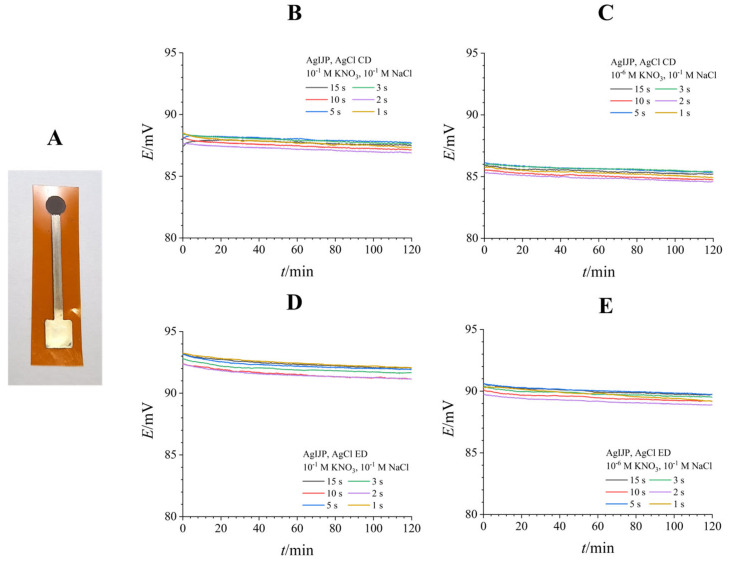
Potential stability testing of chlorinated AgIJPs over 2 h. (**A**)—AgIJP with electrochemically deposited AgCl (10 s). (**B**,**C**)—Chemically deposited AgCl, (**C**,**D**)—Electrochemically deposited AgCl. Stability of chlorinated AgIJPs was tested by varying their ionic strength ((**B**,**D**)—10^−1^ M KNO_3_, (**C**,**E**)—10^−6^ M KNO_3_) at constant chloride concentration (10^−1^ M NaCl).

Figure 1 depicts the inkjet-printed qRE (Figure 1A) and the stability of the AgIJP reference potential in aqueous media of varying ionic strength at constant chloride concentration (Figure 1B–E). Varying the ionic strength has little effect on the reference electrode potential. While both AgIJP-CD (Figure 1B,C) and AgIJP-ED (Figure 1D,E) electrodes exhibit a slight (2–3 mV) drop when switched from 10^−1^ M KNO_3_ to 10^−6^ M KNO_3_, they also show adequate stability over time (potential drifts only 0.3 mV/h). In both cases, an increase in deposition time results in a thicker AgCl layer, which slightly increases potential stability. It is important not to convert all Ag to AgCl, however, as that would result in a nonconductive and ultimately destroyed reference electrode. Therefore, the deposition time was limited to 15 s, as the inkjet-printed Ag layer was a submicron-thick film [40]. Finally, while both chemical and electrochemical deposition produced reference electrodes with satisfactory potential stability, electrochemical deposition resulted in more homogeneous and repeatable AgCl coatings. Ultimately, we settled on a 10 s long electrochemical deposition procedure to ensure the production of repeatable reference electrodes without risking their destruction via overoxidation.

### 3.2. Hydrogen-Selective Electrodes

**Figure 2:**
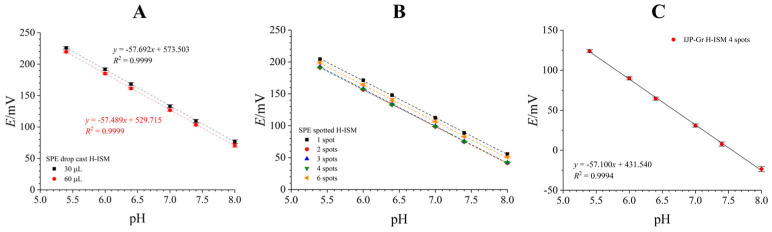
H-ISE prepared on SPEs and IJP-Gr, characterized potentiometrically. (**A**)—SPEs with ISM dropcast on top of them. (**B**)—SPEs with spotted ISMs. (**C**)—IJP-Gr with 4 spots of ISM. All measurements in Figure 2A,B were conducted against an external Ag/AgCl/3M KCl/3M KCl reference electrode. Error bars in (**A**–**C**) represent intraelectrode variability (*n* = 3), while in (**C**) it represents interelectrode reproducibility (*n* = 3).

Ion-selective electrodes with a polymeric membrane employ an ionophore, a compound that reversibly, selectively, and preferably complexes the target ion. As the concentration in the bulk solution increases, the interfacial potential between the sample solution and the ion-selective membrane changes, which is reflected in the potential response of the ion-selective electrode. H-ISEs were prepared with Hydrogen ionophore I as the ionophore and characterized on GCEs (Figure S3A), SPEs, and IJP-Gr (Figure 2). The solvent mixture (50:50 *v*/*v* cyclohexanone and THF) was selected to improve homogeneity of the membrane and minimize the coffee ring [41]. Preliminary testing was conducted by dropcasting the H-ISM onto the working electrode. After drying, the electrodes were conditioned in a phosphate buffer of pH 6.4, after which they were calibrated in phosphate buffer solutions of varying pH levels.

H-ISE prepared by dropcasting (Figure 2A) showed promising Nernstian sensitivity on SPEs, with little difference in sensitivity (57.591 ± 0.144 mV/pH) when comparing different dropcast ISM volumes. These results are comparable to preliminary calibration results of an H-ISM dropcast onto a GCE (Figure S3A). We also prepared a set of spotted H-ISEs by spotting one–six spots of H-ISE onto SPE working electrodes (Figure 2B). Spotting offers a more reproducible and faster fabrication, which can be more easily integrated with the inkjet printing process, therefore reducing the waste of both the electrode and sensing material. No significant differences were found by varying the number of spots on the SPEs. Additionally, spotting provided improved interelectrode E^0^ reproducibility (standard deviation is only 2.56 mV for two–six spots). Therefore, four spots were chosen as the optimal amount for spotting the H-ISM.

Finally, an IJP-Gr was modified with four spots of H-ISM and paired with an AgIJP reference electrode (ED 10 s). Inkjet printing provides several advantages over screen printing, such as minimized waste and reagent use (thus lowering the cost and minimizing the ecological footprint), but it was also found that sensors with improved electrochemical and analytical performance can be obtained by inkjet printing compared to screen printing [42,43]. In solid-contact ion-selective electrodes, graphene is frequently employed as a transducer owing to its excellent processability and favorable physicochemical properties [11,44,45,46]. These include high electrical conductivity, large surface-to-volume ratio, and pronounced hydrophobicity, collectively enabling efficient and well-defined ion-to-electron transduction through a high electrochemical double-layer capacitance. Simultaneously, due to its hydrophobicity, the formation of an undesirable thin water layer at the backside of the ion-selective membrane is efficiently suppressed, thus ensuring stable electrode response during prolonged measurements [14]. Calibration of the H-ISM-modified IJP-Gr resulted in a pH sensor with excellent interelectrode Nernstian sensitivity (57.100 ± 0.727 mV/pH) and linearity (*R*^2^ = 0.9994) through the entire dynamic range (pH 5.4–8), showing promise for application in a flexible format.

### 3.3. Polyaniline Electrodeposition (PANI-ED)

While H-ISE proved successful on the analytical side, there are some biocompatibility considerations to take into account. Membrane components, if leached, could have harmful health effects, especially when it comes to plasticizers and ionophores, which can induce a series of inflammatory responses during in vivo analysis [20]. Therefore, PANI was next evaluated as a non-toxic biocompatible material.

**Figure 3:**
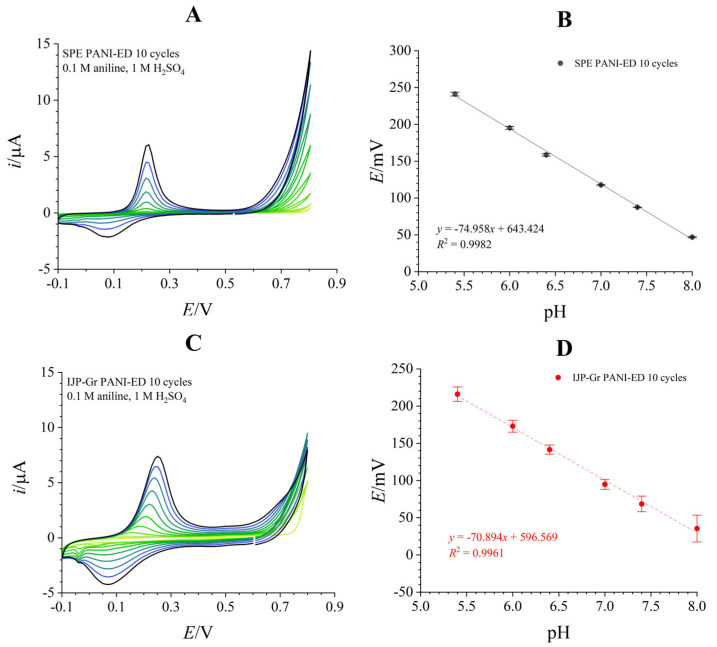
PANI-ED prepared on SPEs (**A**,**B**) and IJP-Gr (**C**,**D**) electrodes. (**A**,**C**)—Cyclic voltammograms showcasing deposition of PANI-ED with each subsequent cycle. (**B**,**D**)—Calibration plots for PANI-ED layers deposited in 10 scans (*n* = 3, intraelectrode reproducibility).

Electrodeposition of PANI is often employed for electrode modification [1,4,26]. PANI-ED coatings were deposited on SPEs and IJP-Gr from a monomer solution by employing cyclic voltammetry according to a procedure found in the literature [4]. Figure 3 shows cyclic voltammograms for the deposition of PANI-ED on SPE (Figure 3A) and IJP-Gr (Figure 3C) electrodes, conducted through 10 scans from −0.1 V to +0.8 V at a potential scan rate of 10 mV/s. The growth of the PANI-ED layer is evident in the increase in peak current magnitudes in the cyclic voltammograms.

The obtained PANI-ED coated electrodes exhibited supernernstian sensitivities with all tested depositions (10–16 cycles), with slopes of ~75 mV/pH for SPEs (Figure 3B and Figure S4) and ~70 mV/pH for IJP-Gr (Figure 3D) coated in this manner. Increasing the number of scans for deposition did not have an effect on sensor sensitivity (Figure S4). A higher sensitivity of electropolymerized PANI (compared to expected Nernstian sensitivity) can be found in the literature, but it is usually bound to a specific pH range or purposefully enhanced using specific dopants or additives [5,22]. However, a sensitivity this high without any specific additives implies that other processes are occurring during the measurement. It is possible that, due to PANI-ED being doped with small sulfate anions, there is a dedoping process occurring simultaneously with the emeraldine salt–emeraldine base pH-dependent switch, as smaller dopants have more mobility for moving out of the PANI structure into the solution [23]. Moreover, while electrodeposition results in homogeneous PANI-ED coatings, which is beneficial from a sensor fabrication viewpoint, aniline electropolymerization by-products were found to be cytotoxic, raising concerns about residuals leaching from an electrodeposited layer [27,47].

### 3.4. Polyaniline Dropcast Deposition (PANI-EB)

Due to the disadvantages of electrodeposition, an alternative method to deposit PANI was tested out: dispersing the PANI-EB powder in a suitable solvent to form a suspension, which can be deposited in various non-contact ways.

**Figure 4:**
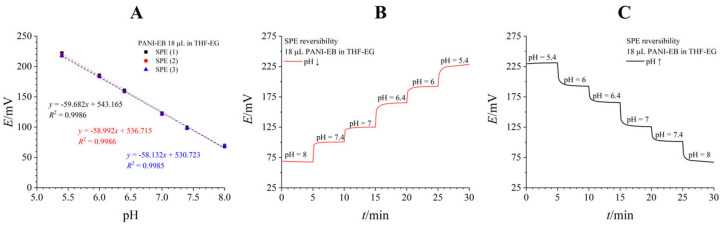
PANI-EB dropcast on SPEs from a THF-EG suspension. (**A**)—Three consecutive calibration curves on the same electrode. (**B**,**C**)—reversibility measured by measuring the pH of standard solutions of decreasing (**B**) or increasing (**C**) pH value.

A mixture of THF and EG in 90:10 *v*/*v* proved to be the best dispersion matrix for our application. THF is a volatile solvent that quickly evaporates, while EG increases viscosity and prevents the solvent from creeping away from the working electrode area. A PANI-EB dispersion in THF-EG was dropcast onto the working SPE. Each layer was dried for at least 10 min until a final dropcast volume of 18 µL was deposited. Figure 4A shows calibration results for three equally modified SPEs, revealing a near-perfect Nernstian sensitivity (58.935 ± 0.777 mV/pH) with remarkable interelectrode reproducibility (*E*^0^ RSD = 1.16%). Figure 4B,C show the potentiodynamic results from reversibility testing in both directions, demonstrating excellent reversibility.

**Figure 5:**
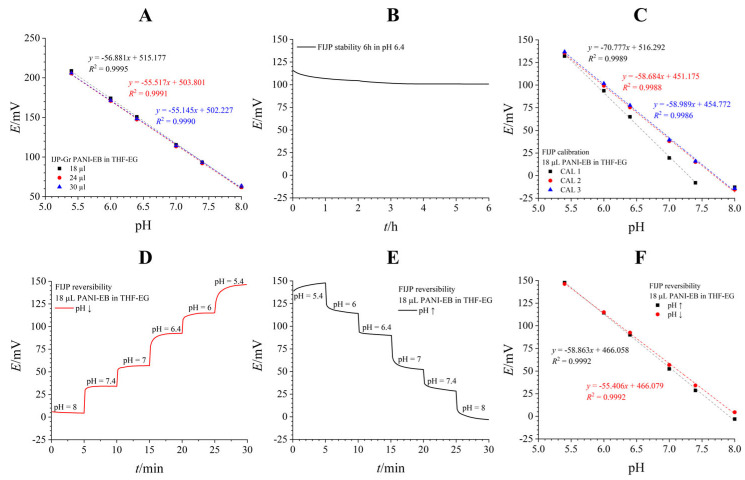
PANI-EB modified IJP-Gr electrodes. (**A**)—Different amounts of PANI-EB aliquots were tested, with little effect on the sensitivity of the electrodes. (**B**)—Potential stability of a FIJP system modified with 18 µL of PANI-EB during 6 h immersion in pH 6.4 buffer solution. (**C**)—Consecutive calibrations on the same FIJP system. (**D**–**F**)—Reversibility of the FIJP system.

After optimizing the PANI deposition method on SPEs, further optimization of the IJP-Gr system was conducted. The amount of the dropcast PANI-EB was taken into consideration, but increasing the dropcast amount had an insignificant and slightly adverse effect on the sensitivity, while barely affecting the calibration curve itself (Figure 5A). Therefore, 18 µL was kept as the optimal dropcasting amount.

Next, the pH-sensitive IJP electrode was paired with an AgIJP electrode coated with AgCl electrochemically. This enabled the use of inkjet-printed electrodes printed in pairs and modified for pH sensing, creating a fully inkjet-printed (FIJP) flexible platform with a biocompatible sensing layer. The FIJP system showed excellent potential stability during 6 h immersion in a buffer solution (pH = 6.4), with a small potential drift of 0.02 mV/h after the initial stabilization period (Figure 5B). Long-term stability testing over a 30 h period was also conducted (Figure S5). After the initial stabilization, the signal remained completely stable during the whole period, with only a minor drift of 0.022 mV/h, which agreed very well with the drift during short-term testing, indicating that the system behaved the same way across different time periods. After short-term stability testing, calibration was repeated with the same electrode with a negligible change in sensitivity. Employing the FIJP system for calibration shows that Nernstian sensitivity is maintained, although prior conditioning of the electrode might be necessary, as evident in the first calibration series in Figure 5C, after which the electrodes provide a stable response with Nernstian sensitivity (58.837 ± 0.216 mV/pH) (Figure 5C).

To validate the pH response in the presence of interferents (such as Na^+^, Mg^2+^, NH_4_^+^, SO_4_^2−^ and NO_3_^−^ [1,4,27]), interference testing (Figure S6) was conducted according to a literature example [4] by adding concentrated interferent solution to achieve final interferent concentrations of 1 mM, 5 mM, and 10 mM, while measuring the open circuit potential of the system at constant pH value (6.4) and chloride concentration (0.2 M KCl). No significant change was observed except for in Mg^2+^ ions, which showed slight interference, evident as a small increase in measured potential. The presence of other interferents did not contribute to the potential response (which was more susceptible to an otherwise present potential drift). Such an observation is in accordance with other results found in the literature [1,4].

Finally, reversibility of the FIJP PANI-EB system was tested by recording calibration curves from lower to higher pH (Figure 5D) and then in the opposite direction (Figure 5E). Minimal hysteresis was observed in the calibration plots when the pH change order was reversed, and the FIJP system exhibited satisfactory Nernstian sensitivity in both directions (Figure 5F). Recovery information (in % error) was calculated from data obtained in Figure 5D–F and is shown in Table S1. The error was under 1% when measuring from higher to lower pH, while somewhat higher when measuring from lower to higher pH (with a maximum 6.90% deviation from the true pH value of 5.4), but for the middle range of pH values, which is also clinically more significant, the error is around 5%.

### 3.5. Proof of Concept: Lung-on-a-Chip

**Figure 6:**
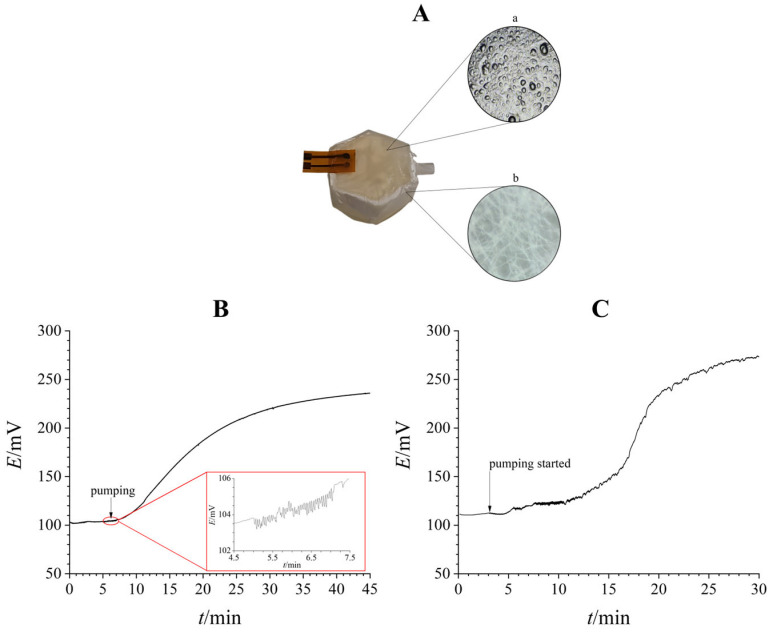
Lung-on-a-chip measurement. (**A**)—Photo of a lung-on-a-chip model with the FIJP system, optical micrography of hydrogel (a) and electrospun membrane (b). (**B**)—pH detection in stationary mode. The inset shows the potential signal while the system is “breathing”. (**C**)—pH detection in dynamic mode.

To test the applicability of FIJP modified electrodes, we integrated them into an artificial lung-on-a-chip model. The lung-on-a-chip housing was designed (Figure S1) based on a detailed analysis of alveolar morphology, particularly from histological sections, which revealed that alveolar units often organize into repeating hexagonal patterns [48]. This natural geometric arrangement, characteristic of the alveolar network, provided the blueprint for the chip’s external structure, but was intentionally scaled up relative to a single alveolus. The resulting 3D-printed housing (Figure 6A) provides a standardized and reproducible platform for in vitro studies of inhaled formulations and bioactive surfaces at the scale of a single alveolar unit. For this design, a nanofibrous PCL membrane was selected due to its ability to simultaneously provide mechanical resilience and a biologically relevant architecture. In the human alveolus, each breath stretches and relaxes an extremely thin barrier that separates air from blood [38]. Recreating this dynamic interface in vitro requires a material that can withstand repeated deformation while offering a structure that cells can recognize and interact with. Electrospun PCL fulfills both requirements, as it forms a mechanically robust yet flexible membrane capable of sustaining cyclic stretching, and its nanofibrous network closely mimics the extracellular matrix, providing a porous scaffold that supports cell adhesion, spreading, and formation of a functional alveolar-capillary barrier. In this context, the PCL membrane functions as both the structural “muscle” and the biological “stage” of the lung-on-a-chip, capable of enduring breathing-like motions while supporting physiologically relevant tissue architecture [49]. Using optical microscopy, the diameter of the electrospun fibers was evaluated and found to reach up to 20 µm (Figure 6A).

Sodium alginate, a naturally derived polysaccharide from brown seaweed, is highly valued for its biocompatibility, non-toxicity, and ability to form hydrogels via ionic crosslinking. These characteristics make it particularly suitable for lung-on-a-chip applications, where a soft, hydrated, extracellular matrix-like environment is required to replicate the mechanical behavior and permeability of alveolar tissue [50]. Optical microscopy revealed that the hydrogel was porous, with pore diameters ranging from 10 to 100 µm, closely resembling the structure of human lung tissue and providing an environment conducive to molecular transport and potential cell integration [51]. Alginate hydrogels combine tunable stiffness, high water content, and mild gelation conditions, enabling the recreation of delicate, compliant lung structures without introducing cytotoxic chemicals or harsh processing steps. Within the context of a lung-on-a-chip system, this hydrogel functions as a supportive but biologically gentle matrix that can emulate the mechanical and transport properties of alveolar tissue [52].

Together, the housing, PCL membrane, and alginate hydrogel created a lung-on-a-chip platform that balanced mechanical resilience, structural fidelity, and physiological relevance. We lastly integrated the FIJP pH-sensor and connected the system to a breathing source to monitor proton diffusion and evaluated the permeability of the alginate-covered electrospun PCL membrane. For this stage of model testing, the permeability of the assembled model was examined by tracking pH change after pumping 10 wt.% HAc every three seconds (Figure 6). A demonstration video of the experiment is available in the Supplementary Materials (Video S1). Two different modes were tested: a stationary mode (initial pumping period of 1 min with a 0.3 Hz frequency followed by no further impulse, Figure 6B) and a continuous “breathing” mode (continuous pumping through 30 min at 0.3 Hz, Figure 6C). Active pumping of 10 wt.% HAc resulted in a signal with visible but small oscillations (Figure 6B inset) around a stable value (standard deviation of 0.458 mV, compared to just 0.147 mV without pumping). The alginate hydrogel exhibited slightly alkaline properties (pH~8), and the results in both cases showed a signal reminiscent of a titration curve. As expected, breathing affected diffusion, and a stable signal was obtained after around 25.5 min in the breathing mode, whereas it took over 36 min for the system to stabilize in the stationary mode. The total potential change of 133 mV in the case of the stationary mode and 169 mV in the case of the continuous breathing mode corresponds to a ΔpH of 2.26 and 2.87, respectively, when a sensitivity of 58.8 mV/pH is taken into account.

## 4. Conclusions

Planar, flexible printed electrodes were successfully modified with pH-sensitive polymeric coatings (H-ISM and PANI) to produce miniaturized potentiometric pH-sensitive electrochemical sensors. Screen-printed and inkjet-printed electrodes with spotted PVC-based H-ISM both exhibited near-perfect Nernstian sensitivity (57.100 ± 0.727 mV/pH for IJPs and 57.551 ± 0.400 mV/pH for SPEs, respectively). Electrodes with PANI-ED coatings exhibited super-Nernstian response, with approximate sensitivities of 75 mV/pH and 71 mV/pH achieved for SPEs and IJP-Gr, respectively. On the other hand, PANI-EB dropcast modification resulted in pH-sensitive electrodes with near-ideal Nernstian sensitivity. Integrated with an inkjet-printed qRE, FIJP systems modified with PANI-EB exhibited excellent sensitivity (58.837 ± 0.216 mV/pH), stability over 6 h and 30 h, reversibility with small hysteresis, and minimal interference from common electrolytes. Finally, the FIJP pH-sensitive system was paired with a lung-on-a-chip system to test its permeability, proving its applicability for biomedical research purposes.

Building on the demonstrated capability of our lung-on-a-chip platform to provide reliable, real-time pH readouts, we aim to further develop the concept towards practical testing of marketed inhalation drugs and future candidate molecules. In addition to potentiometric sensors, we will integrate voltammetric sensors with this function. With multiple sensors, we will look toward role-switching electrode designs, where a single electrode can be sequentially reassigned as a working, counter, or reference electrode for different sensing functions, enabling multimodal analysis with far fewer electrodes [53]. A key advantage of our approach is the complete integration of the pH probe within the hydrogel-based medium, enabling the continuous monitoring of local microenvironmental changes directly at the cell-relevant interface during drug exposure. Such sensor-enabled monitoring is expected to strengthen the predictive value of in vitro inhalation studies by linking formulation- and particle-dependent behavior with time-resolved changes in the local chemical environment.

## Data Availability

Data will be made available from the authors upon request.

## Supplementary Materials

The following supporting information can be downloaded at https://www.mdpi.com/article/10.3390/bios16010038/s1, Figure S1: 3D model of a lung-on-a-chip hexagonal housing; Figure S2: Schematic of the experimental setup used for lung-on-a-chip measurement; Figure S3: GCE measurement preliminary results. A—H-ISM calibration (30 µL drop-cast), B—PANI-ED calibration (deposited through 10 scans); Figure S4: PANI-ED calibrations on SPE with variable number of scans used for deposition; Figure S5: Long-term stability testing; Figure S6: Interference test results; Table S1: Recovery information in % calculated from the reversibility test shown in Figure 5D–F; Video S1: Operation of the lung-on-a-chip system.

## Abbreviations

The following abbreviations are used in this manuscript:

AgIJP	Silver inkjet-printed electrode
AgIJP-CD	Silver inkjet-printed electrode with chemically deposited silver chloride
AgIJP-ED	Silver inkjet-printed electrode with electrochemically deposited silver chloride
DCM	Dichloromethane
DMF	Dimethylformamide
DOS	Bis(2-ethylhexyl) sebacate
EG	Ethylene glycol
FIJP	Fully inkjet-printed
GCE	Glassy carbon electrode
HAc	Acetic acid
H-ISE	Hydrogen-selective electrode
IJP	Inkjet-printed
IJP-Gr	Inkjet-printed graphene
ISE	Ion-selective electrode
ISM	Ion-selective membrane
KTpClPB	Potassium tetrakis(4-chlorophenyl)borate
PANI	Polyaniline
PANI-EB	Polyaniline emeraldine base
PANI-ED	Electrodeposited polyaniline
PANI-ES	Polyaniline emeraldine salt
PCL	Polycaprolactone
PVC	Polyvinyl chloride
qRE	Quasireference electrode
RSD	Relative standard deviation
SPE	Screen-printed electrode
THF	Tetrahydrofuran
