# Supplementary material for: Flexible Inkjet-Printed pH Sensors for Application in Organ-on-a-Chip Biomedical Testing
Source: Biosensors (Basel). 2026 Jan 3;16(1):38. doi: 10.3390/bios16010038 (PMC12839344; doi:10.3390/bios16010038)
Supplement: Supplementary file 1 [file biosensors-16-00038-s001.zip › Bocek PANI LoC SI.pdf]

Flexible Inkjet-Printed pH Sensors for Application in Organ-on-a-Chip Biomedical Testing

Supplementary materials

Željka Boček [1], Donna Danijela Dragun [1], Laeticia Offner [1,2], Sara Krivačić [1], Ernest Meštrović [1,*] and Petar Kassal [1,*]

[1] Faculty of Chemical Engineering & Technology, University of Zagreb, Trg Marka Marulića 19, 10000 Zagreb, Croatia

[2] École Nationale Supérieure de Matériaux, d'Agroalimentaire et de Chimie (ENSMAC), Bordeaux INP, 16 Avenue Pey-Berland, 33607 Pessac, France

Correspondence: emestrov@fkit.unizg.hr (E.M.); pkassal@fkit.unizg.hr (P.K.)

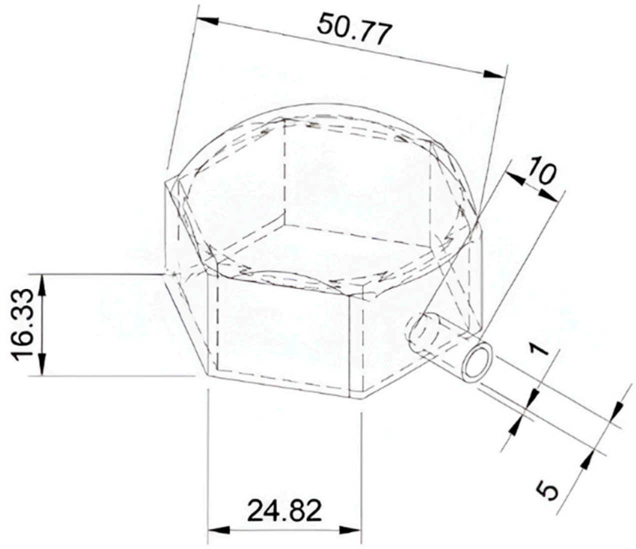

Figure S1. 3D model of a *lung-on-a-chip* hexagonal housing

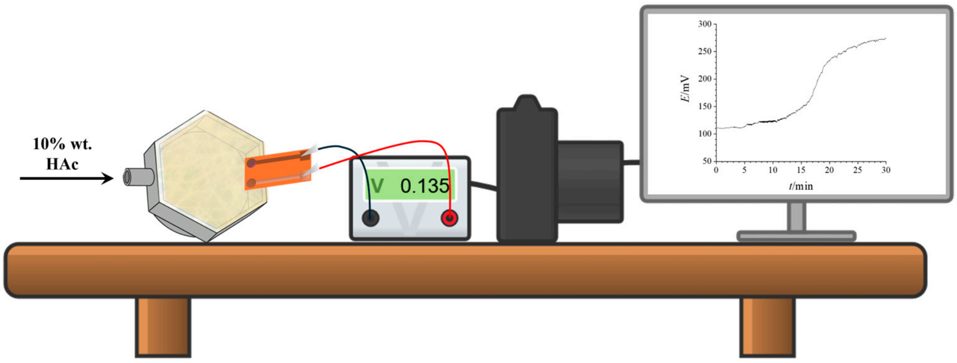

Figure S2. Schematic of the experimental setup used for lung-on-a-chip measurement (created with Chemix (https://chemix.org))

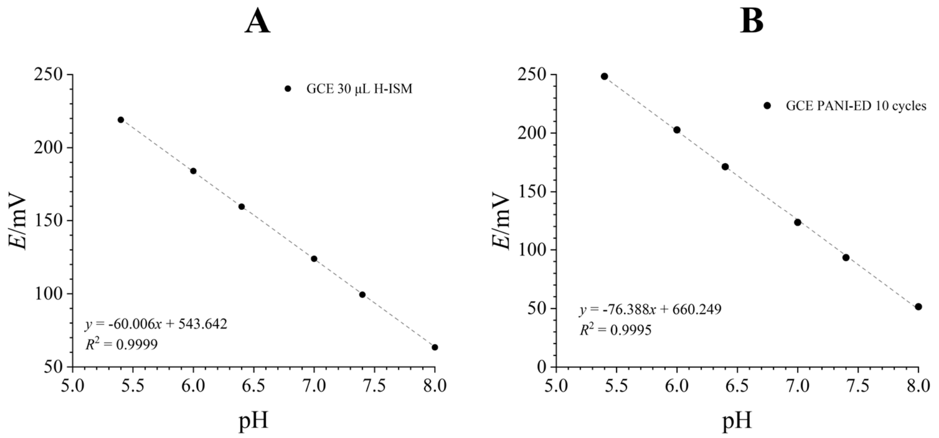

Figure S3. GCE electrode measurement preliminary results. A – H-ISM calibration (30 µL drop-cast), B – PANI-ED calibration (deposited through 10 scans)

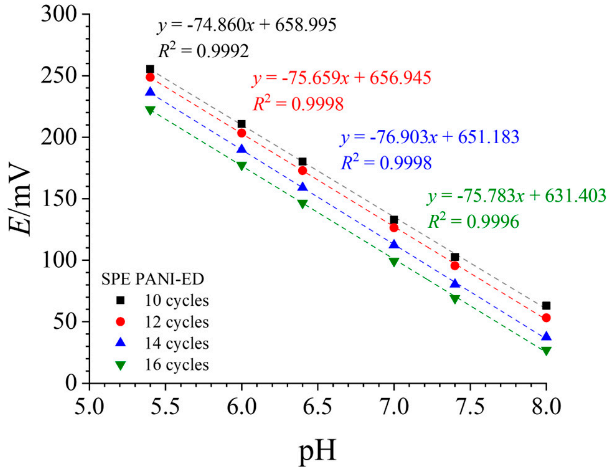

Figure S4. PANI-ED calibrations on SPE with variable number of scans used for deposition

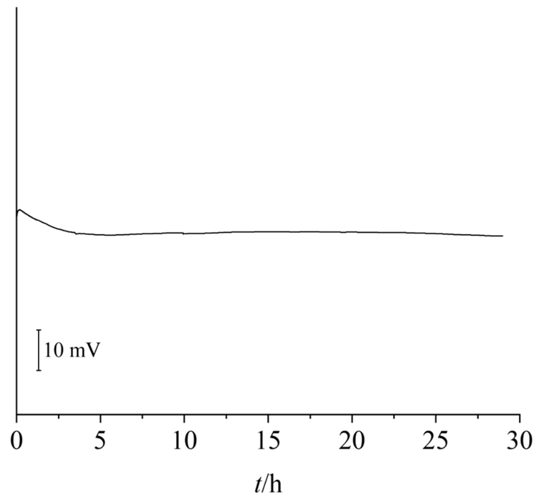

Figure S5. Longterm stability measurement of FIJP system recorded over 29 hours.

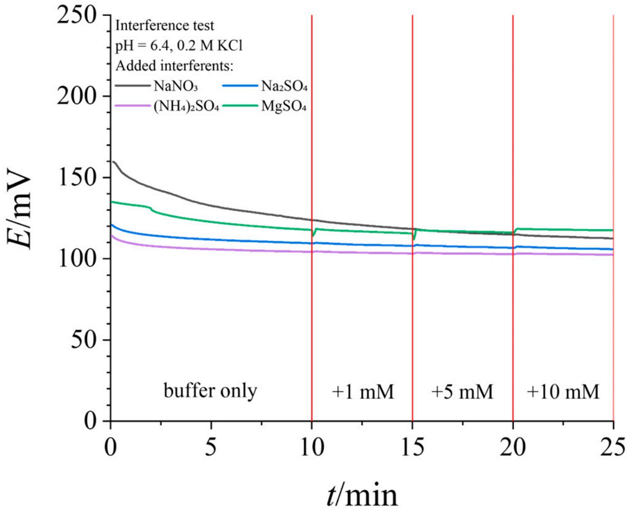

Figure S6. Interference test result.

Table S1. Recovery information in % calculated from the reversibility test shown in Figure 4D-F.

True pH value	Measured pH↑	Error pH↑/%	Measured pH↓	Error pH↓/%
5.4	5.77	6.90	5.41	0.17
6.0	6.34	5.61	5.97	0.42
6.4	6.74	5.36	6.39	0.19
7.0	7.39	5.53	7.03	0.39
7.4	7.79	5.35	7.43	0.43
8.0	8.33	4.13	7.97	0.38
